# RNA Editing and Its Molecular Mechanism in Plant Organelles

**DOI:** 10.3390/genes8010005

**Published:** 2016-12-23

**Authors:** Mizuho Ichinose, Mamoru Sugita

**Affiliations:** 1 Center for Gene Research, Nagoya University, Chikusa-ku, Nagoya 464-8602, Japan; ichinose@gene.nagoya-u.ac.jp; 2 Institute of Transformative Bio-Molecules, Nagoya University, Chikusa-ku, Nagoya 464-8602, Japan

**Keywords:** RNA editing, chloroplasts, mitochondria, plant organelles, C-to-U editing, U-to-C editing, pentatricopeptide repeat (PPR) protein, site-recognition specificity factor, cytidine deaminase

## Abstract

RNA editing by cytidine (C) to uridine (U) conversions is widespread in plant mitochondria and chloroplasts. In some plant taxa, “reverse” U-to-C editing also occurs. However, to date, no instance of RNA editing has yet been reported in green algae and the complex thalloid liverworts. RNA editing may have evolved in early land plants 450 million years ago. However, in some plant species, including the liverwort, *Marchantia polymorpha*, editing may have been lost during evolution. Most RNA editing events can restore the evolutionarily conserved amino acid residues in mRNAs or create translation start and stop codons. Therefore, RNA editing is an essential process to maintain genetic information at the RNA level. Individual RNA editing sites are recognized by plant-specific pentatricopeptide repeat (PPR) proteins that are encoded in the nuclear genome. These PPR proteins are characterized by repeat elements that bind specifically to RNA sequences upstream of target editing sites. In flowering plants, non-PPR proteins also participate in multiple RNA editing events as auxiliary factors. C-to-U editing can be explained by cytidine deamination. The proteins discovered to date are important factors for RNA editing but a *bona fide* RNA editing enzyme has yet to be identified.

## 1. Introduction

RNA editing is a posttranscriptional modification to nuclear, mitochondrial or chloroplast genome-encoded transcripts, and occurs in a wide range of organisms. It was discovered in 1986 in *Trypanosoma brucei* where uridines were inserted at specific sites in the mitochondrial (kinetoplast) cytochrome *c* oxidase II (*coxII*) transcript to restore the proper protein-coding sequence [1], followed by a report that described deletion of uridines in *coxIII* mRNA [2]. This process required guide RNAs encoded by kinetoplast genomes [3]. Similarly, mitochondrial RNAs in the slime mold *Physarum polycephalum* are heavily edited by the insertion of mononucleotides and dinucleotides at specific sites [4]. In addition, A deletions and nucleotide conversions have also been reported. Unlike U-insertion/deletion in kinetoplasts, nuclear-encoded transcripts have been shown to undergo different types of editing; e.g., conversion of cytidine to uridine (C-to-U) in apolipoprotein-B48 mRNA in human and rabbit intestines [5] and adenosine (A)-to-inosine (I) editing in case of GluR-B mRNA encoding a glutamate receptor B of glutamate-gated channels [6]. A-to-I editing has also been reported for several other animal pre-mRNAs. For insights into various types of RNA editing and their respective mechanistic aspects refer to other review articles in this issue.

In the plant kingdom, RNA editing was first identified as a C-to-U exchange in mitochondrial transcripts in 1989 [7,8,9], followed by its reporting in chloroplasts, two years later [10]. RNA editing occurs mostly in translated regions of organelle mRNAs, and occasionally, also in the untranslated regions, introns and structural RNAs [11,12]. Most of the C-to-U changes in the protein-coding region lead to preservation of evolutionarily codons. In some plant taxa, U-to-C “reverse” editing has also been frequently reported in both plant organelles. Therefore, RNA editing is believed to serve as a correction mechanism at the post-transcriptional level for T-to-C (or C-to-T) mutations, probably acting as buffer to less favored mutations in the genomic coding sequences [11,12]. Across plant kingdom, the frequency of organellar RNA editing varies from zero to thousands of sites. No editing seems to occur in nuclear genome-encoded transcripts in plants. Comprehensive and excellent review articles on plant RNA editing have recently been published and describing its mechanistic and functional aspects [13,14,15,16,17]. Here, we briefly summarize the RNA editing events in green plant lineages and current knowledge of *trans*-acting factors involved in C-to-U RNA editing in chloroplasts and plant mitochondria.

## 2. RNA Editing Events in Plant Organelles

### 2.1. C-to-U RNA Editing

RNA editing sites in translated regions can be predicted by a comparison of amino acid sequences deduced from genomic DNA sequences from various plant species. Subsequently, RNA editing can be verified by cDNA sequence analysis. A number of editing sites identified in various land plant mitochondria and chloroplasts [18,19,20,21,22,23,24,25,26,27,28,29,30,31,32,33,34,35,36,37,38,39,40,41,42,43] are listed in Table 1. There are 20 to 60 editing sites in chloroplasts and 300 to 600 sites in mitochondria of most flowering plants, except for the early-branching flowering plant *Amborella trichopoda*. In seed plants, all these editing events are of C-to-U type. Most of the sites in translated regions are efficiently edited, with 90%–100% efficiency, in green leaves. On the other hand, the efficiency of C-to-U editing events that create a translation initiation codon (by an ACG to AUG change) has been surprisingly low. For instance, the editing efficiency at the ndhD-1 site in the *Arabidopsis* chloroplast *ndhD* transcript is 45% [21] and that of the rps14-C2 site in the moss *Physcomitrella patens* chloroplast *rps14* mRNA is 70% in filamentous protonemata, which reduces further to only 20% in leafy tissues [30]. This suggests that editing at this site may regulate translation in chloroplasts. RNA editing efficiency varies in different tissues and organs, developmental stages, or different mutant lines [44].

Recent high-throughput RNA-seq analyses have revealed minor RNA editing events in untranslated regions and intron sequences as well as in protein-coding regions. For instance, in addition to the 34 already known editing sites in Arabidopsis chloroplasts [45], nine novel sites have been identified that are edited at a low level (5% to 12%) [21]. Among the 635 identified editing sites in *Nicotiana tabacum* mitochondria, five sites are in tRNAs and 73 in non-coding regions [38]. Across the plant kingdom, the total number of C-to-U editing sites in chloroplasts varies from 0 in the liverwort *Marchantia polymorpha* to 3415 in the spike moss *Selaginella uncinata* [28] (Table 1).

Out of 3415 sites identified in 74 *S. uncinata* chloroplast mRNAs, 428 are silent editing events, 74 have been identified in four group II introns, 52 create start codons and 31 create stop codons [28]. A total of 2139 editing sites in 18 mRNAs were identified in *S. moellendorffii* mitochondria [41]. Of these, 424 are silent, whereas the others result in 1488 codon changes. In addition, 13 sites are in the two rRNAs [41]. To date, RNA editing sites can be predicted by Plant RNA-editing prediction and analysis computer tools PREPACT 2.0 [46] and PREP-Mt [47]. Some 1800 C-to-U editing sites have been predicted in the *S. moellendorffii* chloroplast, 460 sites in the quillwort *Isoetes flaccida* (chloroplast) and 340 sites in *Huperzia lucidula* chloroplasts [28]. Therefore, the organellar transcripts in *Selaginella*, one of the early vascular plant lycopods, seem to be most commonly edited.

In case of the bryophyte (early non-vascular land plants) *P. patens*, where there are only two identified C-to-U editing sites in chloroplasts [30], there are 11 such site in its mitochondria [42,43]. On the other hand, hornworts such as *Anthoceros* and *Phaeoceros laevis* undergo substantial RNA editing [29,48]. However, no editing event has so far been reported in green algae, including *Chara vulgaris* (stonewort), suggesting that the process of RNA editing may have evolved only after the plants established themselves on the land.

### 2.2. U-to-C RNA Editing

Although in none of the seed plants listed in Table 1, U-to-C RNA editing has been reported in either of the two organelles, a 25 year old report describes U-to-C editing in plant mitochondria in wheat *cox3*, evening primrose *cob* and *cox2* and pea *cox2* transcripts (references in [11]). While these data need to be reanalyzed, the rare instances of reverse-type editing occurring in seed plants cannot to completely ruled out. In contrast to higher plants, the reverse (U-to-C) editing appears to be restricted to hornworts, lycophytes, and ferns [27] with an extensive array of (over 400) U-to-C editing sites identified in the hornwort, *Anthoceros angustus*, chloroplasts [29] and mitochondria of two other hornwort species [48] as well as in the early vascular plant lycophyte *Isoetes engelmannii* [40]. The cDNA sequence analysis of four selected mitochondrial genes from nearly 30 ferns species has revealed that both types of editing is prevalent in most fern species, and notably, the reverse (U-to-C) editing could even exceed the C-to-U RNA editing in some ferns [27]. For instance, 53 C-to-U and 70 U-to-C editing sites were detected in the 1020 bp cDNA sequence of the mitochondrial *atp1* gene of *Anemia phyllitidis*. In other ferns such as *Equisetum hyemale*, several C-to-U editing sites but no U-to-C editing have been found in the selected gene transcripts. In *E. hyemale* chloroplasts, the RNA editing is completely absent [27]. It would be interesting to know the reasons for the expansion of U-to-C type of editing only in some specific plant taxa, including the Monilophytes. The evolution of editing in Monilophytes spans a much longer timeframe, probably as old as the seed plants. Hornworts, some lycopytes, and ferns produce spores but not seeds. It is possible that seed plants and some bryophytes might have lost the U-to-C editing during the course of evolution.

## 3. RNA Editing Affects tRNA Maturation and RNA Splicing

RNA editing in plant organelles mostly affects mRNAs, thus providing the means to correct genetic information for proper protein function. In addition, editing affects some tRNAs and rRNAs encoded in the organellar genomes [49,50,51,52]. In bean and potato mitochondria, a C-to-U editing event corrects a C:A mismatch base pair into a U:A base pair in the acceptor stem of tRNA^Phe^ [51]. In larch, three C-to-U editing events restore U:A base pairs in the acceptor, D and anticodon stem, respectively, in mitochondrial tRNA^His^ [52]. In the lycophyte *I. engelmanni* mitochondria, ten tRNAs are edited to improve base pairing in stem regions [40]. Thus, editing events in pre-tRNAs help in restoring the RNA secondary structure by removing mismatches in the double-stranded stem region and are a prerequisite for their processing into functional tRNAs.

Exemplifying the rarity of editing in rRNAs in seed plants, there was no such report for a long time after an initial reporting of two potential sites in *Oenothera* mitochondrial 26S rRNA 25 years ago [50]. Recently however, 13 C-to-U editing sites have been identified in two rRNAs in the lycophyte *S. moellendorffii* mitochondria [41]. Three of these sites are in the 26S rRNA, and rest 10 are in the first exon of 18S rRNA. Notably, RNA editing at the last nucleotide of the 18S rRNA 5′ exon may directly influence splicing of its group I intron, as it likely forms the U:A base pairing needed for the conserved paired region P1 [41].

Like some instances in tRNAs, perhaps the editing sites within group II introns are also of functional importance because editing can improve the base pairing required for splicing. Domain VI of *nad1* group II intron 3 from *Oenothera* mitochondria is modified by C-to-U editing to generate the typical domain VI secondary structure. Self-splicing in vitro is observed only in the edited (A:U basepair) form, indicating that this editing event is a prerequisite for splicing [53]. In the lycophyte *S. uncinata* chloroplasts, a number of intron editing events have been identified, which could possibly improve the RNA secondary structure of group II introns, including the highly conserved domains V of the intron 3′ termini [28]. Such editing events could potentially play significant role in splicing, thereby regulating the availability of functional tRNAs.

RNA editing in exons close to splice sites may also affect intron splicing or vice versa. For instance, the spinach chloroplast *ndhA* mRNA is edited at two sites, one of which is located only 12 nucleotides downstream of the 3′ intron-exon splice site. To assess if RNA editing occured after or before splicing, short “spliced” and “unspliced” *ndhA* gene fragments were introduced and transcribed within tobacco chloroplasts. The subsequent cDNA analysis showed that only spliced *ndhA* mRNAs were edited [54]. A similar result was observed in case of the moss *P. patens* mitochondrial *atp9* gene [55]. This *atp9* gene is interrupted by three introns and an editing site lies within the third exon (only 8 nt long). This site is completely edited in fully spliced mRNA, while it remains unedited in the unspliced mRNA [55]. These observations suggest that splicing precedes editing. In contrast, the land plant chloroplast tRNA^Leu^ gene contains a group I intron between the first and second position of the UAA anticodon. In the moss *Takakia lepidozioides*, the CAA anticodon of tRNA^Leu^ is edited to create a canonical UAA codon [56]. The spliced tRNA^Leu^ is completely edited while unspliced tRNAs are partially edited. This suggests that the anticodon editing of tRNA^Leu^ may occur before RNA splicing.

## 4. RNA Editing Factors in Plant Organelles

### 4.1. PPR Proteins as a Site-Recognition Factor

**Figure 1:**
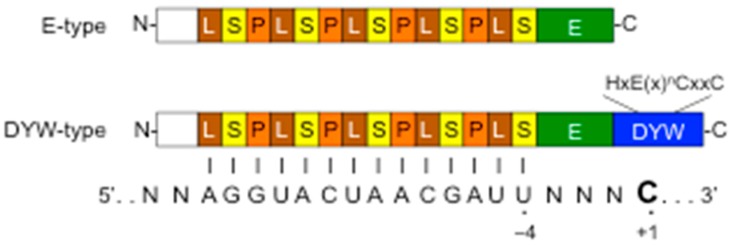
Plant organellar pentatricopeptide repeat (PPR) editing proteins and a model for their binding to the editing site. Schematic domain structure of PPR editing proteins that consist of PPR motifs (P, L, S), and additional C-terminal domains (E and DYW). The DYW domain contains the conserved zinc-binding motif signature, HxE(x)^n^CxxC. The PPR tract interacts with a target RNA in a one PPR motif to one nucleotide manner. The last PPR S motif recognizes nucleotide at position –4 from the editing site (+1).

To elucidate the molecular mechanism of RNA editing in plant organelles, in vitro, in vivo, and in organello studies have extensively been performed using flowering plants such as wheat, tobacco, pea, and cauliflower [57,58,59,60,61]. These studies have helped in identifying *cis*-acting elements adjacent to editing sites and discovering putative site-specific proteins that interact with these elements. In all such instances, the *cis*-elements comprise stretches of 20 to 25 nucleotides upstream of the editing sites. The identification of the first *trans*-acting factor, however, was not the outcome of any genetic and biochemical study for editing factors, but instead it resulted from a study on photosynthetic mutants in 2005 [62]. One of the isolated mutants, *chlororespiratory reduction 4* (*crr4*), showed defects in the accumulation of the plastidic NADH dehydrogenase (NDH) complex, which is a multi-subunit complex in the thylakoid membrane. The loss of NDH complex was correlated directly to the loss of a C-to-U editing event that otherwise creates the start codon AUG in *ndhD* mRNA. It was later found that the CRR4, a member of the pentatricopeptide repeat (PPR) protein family, binds to a 36 nucleotides (−25 to +10) region surrounding its target editing site [63]. This suggested that CRR4 could be the *bona fide trans*-acting factor essential for recognizing this RNA editing target site. Following this discovery, several other PPR proteins were identified as site recognition factors affecting editing in chloroplasts and mitochondria [64,65] (Figure 1). Many editing PPR proteins were found to be responsible for only a single editing site, whereas, some PPR proteins could recognize multiple sites with similar *cis*-element sequences [13,14,15,16,17].

PPR proteins constitute a large family of nuclear-encoded proteins comprising of 100 to over 1000 members in land plants [66,67,68]. However, there number varies from only several to 20 members in fungi, protists, and animals [66]. Almost all the PPR proteins are localized in either chloroplasts or mitochondria, or both [69] where these proteins participate in different facets of RNA metabolism such as RNA splicing, RNA stability, and translational initiation [70]. PPR proteins are characterized by tandem arrays of the degenerate 31 to 36-amino acid PPR motif that folds into a pair of anti-parallel alpha helices, which have been suggested to specifically bind to RNA sequence targets [71].

The PPR proteins are structurally divided into two major classes, denoted P and PLS. The P-class is composed of canonical PPR (P) motifs of 35 amino acids, while the PLS-class consists of canonical P motifs and their variants L (for long, 35 or 36 amino acids) and S (for short, 31 amino acids), which differ in sequence length and conservation [66,70]. At their C-terminus, following the last PPR motif, many PLS-class PPR proteins are extended by the plant-specific conserved E (extension) domain, and are thus occasionally called PPR-E or E-type PPR proteins. The Arabidopsis CRR4 also belongs to this category. About half of the PLS-class PPR proteins with the E domain are further extended by a DYW domain of about 100 amino acids and are named after its three highly conserved C-terminal amino acids, aspartic acid (D), tyrosine (Y), and tryptophan (W). The PLS-class PPR proteins are found only in land plant lineages but not in algae and non-plants. Intriguingly, DYW-type PPR proteins were found in the protist microscopic amoeba *Naegleria*, in the slime mold *Physarum* and in the wheel animal *Rotifera* [72,73,74]. To date, nearly 70 PPR editing factors have been identified in seed plants and the moss, *P. patens* [15,16]. All of them belong to the PLS-class with C-terminal E or E-DYW domains (Figure 1).

**Figure 2:**
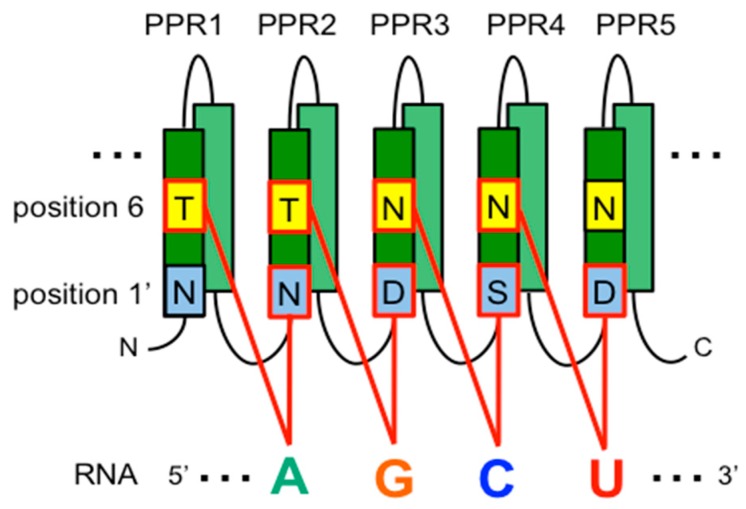
PPR recognition code for RNA binding. Key amino acid positions 6 and 1′ of each PPR motif are indicated as yellow and blue colored square boxes, respectively. T, N, D, and S denote amino acids tyrosine, asparagine, aspartic acid, and serine, respectively. Combinations of amino acids at positions 6 and 1′ specify binding to specific bases as proposed in Barkan et al. [75]. (T, N) (T at 6, N at 1′) specify binding to adenine (A), (T, D) to guanine (G), (N, S) to cytidine (C), (N, D) to uridine (U), and (N, N) to C or U.

Recently, a code for PPR-RNA recognition has been elucidated [75,76,77]. The amino acid combinatorial patterns at position 6 and 1′ (position 1 of the following PPR motif) recognize a specific RNA base (Figure 2). The PPR editing proteins bind to a specific *cis*-element for editing. PPR crystal structure analyses have shown that a PPR in N-to-C terminus orientation interacts with RNA in the 5′ to 3′ orientation for the target RNA and confirms the RNA-binding code [78,79,80,81]. Similarly, PPR editing proteins bind to specific *cis*-elements for editing in a one-PPR motif to one-nucleotide manner.

### 4.2. Importance of the DYW Domain in RNA Editing

Plant-specific E domains, which contain two PPR-like motifs, have been shown to be essential for editing [64,82]. Okuda et al. [83] demonstrated that the DYW domains of CRR28 and OTP85 interact with the target C, whereas the E domain of CRR21 is not involved in binding. The exact role of the E domain in editing remains unclear, it is speculated however that it might be involved in interacting with other proteins.

**Figure 3:**
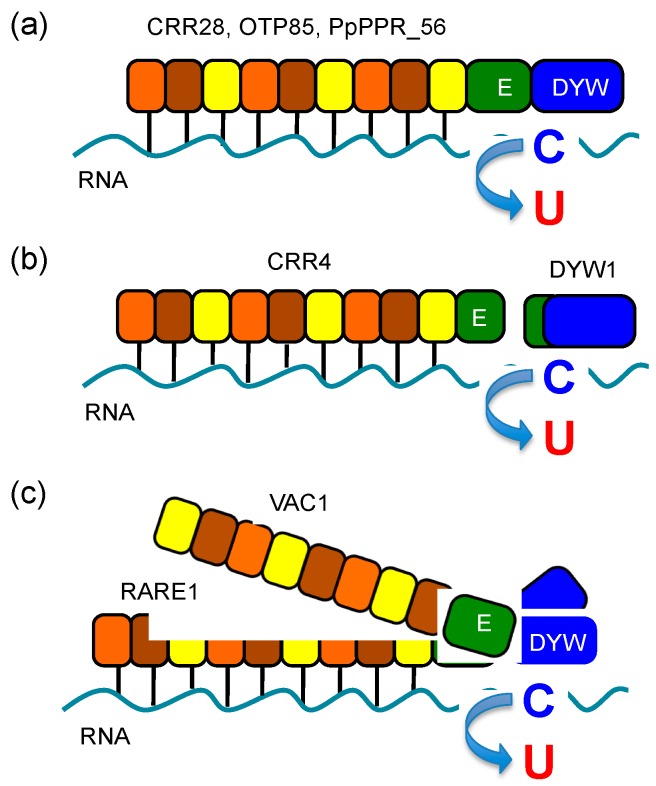
RNA editing requires a single or multiple PPR editing factors. (**a**) Single PPR-DYW editing proteins (e.g., CRR28, OTP85, PpPPR_56) are involved in editing at their target sites; (**b**) PPR-E editing factor (e.g., CRR4) and DYW1 are both required for editing at a single site. PPR proteins recognize the target editing site and DYW1 are involved *in trans* in editing; (**c**) Two PPR-DYW proteins are cooperatively involved in editing. Either of two PPR-DYW proteins is involved in site recognition and another one may be required for the C-to-U editing reaction.

Like E-type PPR proteins, in most cases a single PPR-DYW editing protein is involved in RNA editing at target sites (Figure 3a). The DYW domains contain the canonical zinc-binding motif HxE(x)nCxxC, which is also found in other cytidine deaminases [84]. In addition, there also exists a correlation between the evolutionary distribution of nuclear DYW domains and instances of organelle RNA editing among land plants [85]. Put together, these findings suggest that DYW domain could harbor the cytidine deaminase enzymatic activity. However, the RNA deamination activity of the DYW domain has not yet been proven by any of the studies deploying in vitro editing assay systems [82,86]. Furthermore, genetic analyses carried out on CRR22 and CRR28 suggest that the DYW motif is dispensable for the editing activity [82], further accentuating the ambiguity over the role of the DYW domain.

In contrast to the findings in *Physcomitrella*, the DYW1 protein in *Arabidopsis* has been identified as an RNA editing factor acting specifically on the chloroplast *ndhD*-1 site [87]. DYW1 consists of a partial E domain and a well-conserved DYW domain with no PPR motifs. In chloroplasts, the DYW1 interacts in with an E-type PPR protein CRR4 to edit the ACG codon to an AUG in the *ndhD* mRNA. Both proteins have been shown to be required for this editing event, suggesting that the DYW domain is essential but could be provided in *trans* if not present in *cis* on the PPR editing factor (Figure 3b). There are five other DYW1-like proteins in *Arabidopsis*, suggesting that association with DYW proteins could be a general feature of E-type PPR editing factors [87].

The PPR proteins have also been shown to act cooperatively, as the loss-of-function of one may reduce but not completely abolish editing at a particular site, suggesting that the remaining editing could be carried out by its other counterpart(s). For instance, RARE1 and VAC1 (also called AtECB2), both of which are DYW-type PPR proteins, are identified as editing PPR proteins targeting the same *accD*-C794 site in *Arabidopsis* chloroplasts [88,89]. Mutation of the *RARE1* gene results in a complete loss of *accD* editing [88] while that of VAC1 leads to a 60% reduction of editing compared to the wild type level [89]. An in silico target assignment test suggested that RARE1, but not VAC1, is indeed a site-recognition factor for *accD* editing [90]. VAC1 is involved in *accD* editing, but might not be required for site recognition. These two PPR-DYW proteins could be cooperatively involved in *accD* editing, and VAC1 may interact with RARE1, as DYW1 does with CRR4 (Figure 3c).

Similarly, studies on moss PPR editing factors also support the importance of the DYW domain in RNA editing. The moss (*P. patens*) genome encodes 10 DYW-type PPR proteins but no E-type PPR protein. In this moss, the 13 C-to-U editing events are coordinated by nine DYW-type PPR proteins [55,91,92], implying that one or more PPR-DYW proteins would have to act as a site-recognition factor for more than one editing sites (Figure 3a). This is the first full assignment of nuclear-encoded DYW-type editing protein factors to all its organellar editing sites in a plant species.

Recently, DYW domains have been shown to bind zinc ions and have been implicated in RNA editing in *Arabidopsis* [93,94]. Moreover, in vitro RNA binding assays have shown that DYW domains in chloroplast editing factors, CRR28 and OTP85, directly bind to their target Cs and respective 5′ proximal region from –3 to 0 (+1C) [83]. This suggests their involvement in the C-to-U catalytic reaction. On the same lines, we have also observed that the zinc-binding motif of DYW-type protein PpPPR_56 is essential for editing at two mitochondrial sites *nad3*-C230 and *nad4*-C272 in *P. patens* (Ichinose and Sugita, unpublished). Various transgenes for wild type *PpPPR_56* (56comp), and mutant variants with the HxE(x)nCxxC motif changed to alanine, AxA(x)nCxxC (56M1) and HxE(x)nAxxA (56M2), respectively, were introduced into the *PpPPR_56* knockout moss (∆*56*-22). In the 56comp moss line, editing of *nad3*-C230 and *nad4*-C272 sites was restored to wild type levels. Whereas, 56M1 and 56M2 constructs failed to complement the mutant editing phenotype, thereby suggesting that the zinc-binding motif of the DYW domain could also play an important role in the process of editing in the moss system as well.

### 4.3. Non-PPR Editing Factors in Plant Organelles

Besides RNA sequence-specific PPR editing factors, another group of proteins, known as multiple organellar RNA-editing factors (MORFs) have also been linked to RNA editing in flowering plants [95]. MORFs are also known as RNA editing Interacting Proteins (RIPs) [96]. Ten members of the MORF family, with a novel conserved protein domain, named the MORF domain, have been identified in *Arabidopsis*. Seven of these target sites in mitochondria, two (MORF2 and 9) in chloroplasts and one (MORF8) acts on its targets in both the organelles. In contrast to PPR editing factors, mutants of either *MORF2* or *MORF9* gene are affected at almost all RNA editing sites in *Arabidopsis* chloroplasts [95]. This suggests that editing of the ndhD-1 site requires at least four proteins: CRR4, DYW1, MORF2 and MORF9 [80]. Similarly, mitochondria-localized MORFs are also involved in RNA editing at many sites. MORF proteins have been shown to interact with each other and also with some PPR editing factors [97,98] and form specific homo- and heteromeric interactions [99]. These factors are organized in a higher ordered editing complex (~200 kDa, called the editosome) [96]. Although the actual function of MORF proteins in the editosome in organelles is as yet unknown, the members of this family may act as connectors between the PPR editing factors and the actual cytidine deaminase activity site in the editosome. This hypothesis, however, needs to be validated. The mitochondrial MORF proteins discriminate between different PPR proteins in yeast two-hybrid assays [95]. In some instances, the MORF proteins that are required for editing at a given site indeed interact with the specific PPR protein, which is also essential for processing that particular site. The MORF proteins may be involved in bridging the distance of four nucleotides between the nucleotides contacted by the PPR proteins and the actually edited C moiety to guide the enzyme.

Other types of proteins involved in RNA editing belong to RNA recognition motif (RRM)-containing proteins: chloroplast ribonucleoproteins (cpRNPs) and organelle RRM proteins (ORRMs). CP31A, a member of the cpRNP family containing two RRMs, influences the efficiency of editing at 13 sites in *Arabidopsis* chloroplasts [100]. However, this effect on editing is possibly indirect because the levels of many other transcripts are also reduced in the *cp31a* mutant [101]. The ORRM family proteins have also been shown to be involved in the process of editing in *Arabidopsis* and maize [102,103,104]. ORRM1 is a chloroplast localized protein that is characterized by two truncated MORF domains and one RRM domain [102]. The loss of ORRM1 leads to a drastic reduction of editing at 12 sites in *Arabidopsis* and nine sites in maize chloroplasts. ORRM2, ORRM3 and ORRM4, which have a single RRM domain and a glycine-rich domain, are likewise shown to be important for efficient editing at many mitochondrial sites [103,104]. ORRMs have also been shown to associate with MORF proteins and form homo or heteromeric interactions. In addition, ORRM1 can interact with PPR editing factors. Similar to MORFs, it seems likely that the ORRMs also are major components of the editosome.

Two additional novel proteins, PPO1 (protoporphrinogen IX oxidase 1) and OZ (organelle zinc finger), have been characterized as general editing factors [105,106]. Notably, PPO1, a critical enzyme for the tetrapyrrole biosynthetic pathway, plays an unexpected role in chloroplast editing at multiple sites in *Arabidopsis* [105]. PPO1 interacts with three chloroplast MORF proteins but not with PPR proteins. These data suggest that PPO1 controls the level of chloroplast editing via the stabilization of MORFs. The OZ family contains 4 members, OZ1-4, in *Arabidopsis* of which three are predicted to be localized in chloroplasts while one is mitochondrial. The OZ1 was identified by co-immunoprecipitation with DYW-type PPR editing factor, RARE1 [106]. Disruption of *OZ1* in *Arabidopsis* leads to an alteration in the level of editing of most sites in chloroplasts. OZ1 can interact with PPR editing factors, where it is assigned to the cognate sites, and ORRM1, but not with MORFs. This interaction supports the notion that OZ1 takes part in the editosome of chloroplasts.

## 5. Mechanism of RNA Editing in Plant Organelles

In plant organelles, PLS-type PPR proteins recognize and bind specifically to editing sites. The PPR–RNA complex is organized into the editosome with several additional non-PPR protein factors such as MORF and ORRM proteins. However, the order of addition/assembly of individual protein factors into the editosome has yet to be clarified. These non-PPR proteins might play a key role in editing as regulators of editing efficiency or as connectors of site-specific PPR proteins with other proteins or an unidentified editing enzyme. Presumably, approximately 200 PLS-type PPR proteins found in Arabidopsis might be involved in RNA editing. Henceforth, several of the editing PPR protein would have to recognize more than one editing sites to be able to recognize all 600 editing events in *Arabidopsis*. PPR proteins and non-PPR editing factors are targeted to either chloroplasts or mitochondria, or both. This suggests that the basic machinery for a C-to-U editing event is perhaps conserved in both organelles. This editosome model has been drawn from molecular evidence found mostly in *Arabidopsis*. However, in the early land plants (mosses) and early vascular plants (lycophytes), the non-PPR editing factors described above are not encoded in their nuclear genomes. Unlike the complex editosome of seed plants, RNA editing may occur in a simpler editing complex, composed of a single PPR-DYW editing protein and a few other unidentified non-PPR editing factors, at least in mosses. The remaining central question is the nature of the RNA editing enzyme. Despite circumstantial evidence supporting the DYW editing enzyme [85,86], a biochemical demonstration of cytidine deaminase activity for the DYW domain would be required to prove that DYW indeed is the editing enzyme.

## 6. Conclusions and Perspectives

In some plant species, the existence of both conventional C-to-U and reverse U-to-C editing events is highly evident, but the mechanism of target recognition and features of the editing factors involved are completely unknown. In the fern *A. phyllitidis*, there are 53 C-to-U and 70 U-to-C editing events known in the 1 kb mitochondrial *atp1* mRNA [27]. Such a high density, of more than 100 target sites in a 1 kb transcript, suggests that there would exist several overlapping *cis*-elements which are needed to be properly identified in their unedited, partially-edited or fully-edited states. Although C-to-U editing requires PLS type PPR proteins, it is unclear if the same were true also for the reverse (U-to-C) editing. However, the possibility of PLS-type PPR proteins being involved in reverse editing cannot also be ruled out either. About 6000 C-to-U (but no U-to-C) editing sites are present in some *Selaginella* species, suggesting that thousands of PPR editing factors could be involved in all editing events.

To determine the *cis*-elements for editing, an in vitro or in organello assay system must be developed from the plants in which reverse editing also occurs. However, so far it has been difficult to prepare purified organelles from these plants. To identify the editing factors, a forward and/or reverse genetic approach could be useful, as it has been in case of flowering plants. However, such approaches have yet to be applied. The development of breakthrough technologies for RNA editing studies in reverse-editing plant taxa needs to be established in the near future. As an alternative approach, candidate editing factors could possibly be identified from the enormous genomic data that have been accumulated for bryophytes, lycophytes, and ferns. Subsequently, a loss-of function of those candidates via genome editing might lead to the identification of novel editing factors.

## Figures and Tables

**Table 1:** The numbers of RNA editing sites in chloroplasts and plant mitochondria.

Plant Species (Common Name)	RNA Editing Type		References
	C-to-U	U-to-C	
**Chloroplasts**			
**Seed plants (monocotyledonous angiosperms)**			
*Oryza sativum* (rice)	21	0	[18]
*Zea mays* (maize)	26	0	[19]
*Spirodela polyrhiza* (greater duckweed)	66 *	0	[20]
**Seed plants (dicotyledonous angiosperms)**			
*Arabidopsis thaliana* (thale cress)	43 *	0	[21]
*Nicotiana tabacum* (tobacco)	34	0	[22]
*Cucumis sativus* (cucumber)	51	0	[23]
*Amborella trichopoda*	138	0	[23]
**Seed plant (gymnosperms)**			
*Cycas taitungensis* (Emperor Sago)	85	0	[24]
**Ferns**			
*Adiantum capillus-veneris* (southern maidenhair fern)	315	35	[25]
*Ophioglossum californicum* (California adder’s tongue fern)	297	3	[26]
*Psilotum nudum* (whisk fern)	27	0	[26]
*Equisetum hyemale* (horsetail)	0	0	[27]
**Lycophytes**			
*Selaginella uncinata* (spike moss)	3415 *	0	[28]
**Bryophytes**			
*Anthoceros angustus* (hornwort)	509	433	[29]
*Physcomitrella patens* (moss)	2	0	[30]
*Marchantia polymorpha* (liverwort)	0	0	
**Mitochondria**			
**Seed plants (monocotyledonous angiosperms)**			
*Oryza sativum*	491	0	[31]
**Seed plants (dicotyledonous angiosperms)**			
*Arabidopsis thaliana*	619 *	0	[32,33]
*Brassica napus* L. (rapeseed)	427	0	[34]
*Beta vulgaris* (sugarbeet)	357	0	[35]
*Vitis vinifera* (grapevine)	445 *	0	[36]
*Phoenix dactylifera* L. (date palm)	592	0	[37]
*Nicotiana tabacum*	635 *	0	[38]
**Seed plant (gymnosperms)**			
*Cycas taitungensis*	565	0	[39]
**Lycophytes**			
*Isoetes engelmannii* (Engelmann′s quillwort)	1560 *	222 *	[40]
*Selaginella moellendorffii* (spike moss)	2152 *	0	[41]
**Bryophytes**			
*Physcomitrella patens*	11	0	[42,43]
*Marchantia polymorpha*	0	0	

Numbers of editing sites in species in which full complement have been analysed. * Data from RNA-seq analyses.
